# A thirteen-year analysis of *Plasmodium falciparum *populations reveals high conservation of the mutant *pfcrt *haplotype despite the withdrawal of chloroquine from national treatment guidelines in Gabon

**DOI:** 10.1186/1475-2875-10-304

**Published:** 2011-10-17

**Authors:** Matthias Frank, Nicola Lehners, Pembe I Mayengue, Julian Gabor, Matthias Dal-Bianco, David U Kombila, Ghyslain Mombo Ngoma, Christian Supan, Bertrand Lell, Francine Ntoumi, Martin P Grobusch, Klaus Dietz, Peter G Kremsner

**Affiliations:** 1 Institute of Tropical Medicine, University of Tübingen, Wilhelmstr.27, 72074 Tübingen, Germany; 2 Medical Research Unit, Albert Schweitzer Hospital, Lambaréné, Gabon; 3 Department of Medical Biometry, University of Tübingen, Germany; 4 Infectious Diseases, Tropical Medicine and AIDS, Amsterdam Medical Center, University of Amsterdam, The Netherlands; 5 Congolese Foundation for Medical Research/Faculty of Health Sciences, University Marien Ngouabi, Brazzaville, Congo; 6 Département de Parasitologie, Université des Sciences de la Santé, Libreville, Gabon

**Keywords:** chloroquine resistance, chloroquine sensitivity, microsatellite analysis, haplotype analysis, artesunate plus amodiaquine

## Abstract

**Background:**

Chloroquine resistance (CR) decreased after the removal of chloroquine from national treatment guidelines in Malawi, Kenia and Tanzania. In this investigation the prevalence of the chloroquine resistance (CQR) conferring mutant *pfcrt *allele and its associated chromosomal haplotype were determined before and after the change in Gabonese national treatment guidelines from chloroquine (CQ) to artesunate plus amodiaquine (AQ) in 2003.

**Methods:**

The prevalence of the wild type *pfcrt *allele was assessed in 144 isolates from the years 2005 - 07 by PCR fragment restriction digest and direct sequencing. For haplotype analysis of the chromosomal regions flanking the *pfcrt *locus, microsatellite analysis was done on a total of 145 isolates obtained in 1995/96 (43 isolates), 2002 (47 isolates) and 2005 - 07 (55 isolates).

**Results:**

The prevalence of the mutant *pfcrt *allele decreased from 100% in the years 1995/96 and 2002 to 97% in 2005 - 07. Haplotype analysis showed that in 1995/96 79% of the isolates carried the same microsatellite alleles in a chromosomal fragment spanning 39 kb surrounding the *pfcrt *locus. In 2002 and 2005 - 07 the prevalence of this haplotype was 62% and 58%, respectively. *Pfcrt *haplotype analysis showed that all wild type alleles were CVMNK.

**Conclusion:**

Four years after the withdrawal of CQ from national treatment guidelines the prevalence of the mutant *pfcrt *allele remains at 97%. The data suggest that the combination of artesunate plus AQ may result in continued selection for the mutant *pfcrt *haplotype even after discontinuance of CQ usage.

## Background

The evolution of CQR has been a major obstacle to global public health [1]. CQR reached Africa in the late seventies and reached a high prevalence across the continent over the following decade [2]. Malawi was the first African country to discontinue the use of CQ in 1993 [3]. Interestingly, the prevalence of the resistant *pfcrt *allele 76T decreased drastically from 85% to 13% within the following eight years [3,4]. In 2005, a clinical trial showed a 100% efficacy of CQ for treatment of uncomplicated *Plasmodium falciparum *malaria in children [5]. Recently, Kenya and Tanzania have also reported a decline in CQR after replacement of CQ by sulphadoxine-pyrimethamine (SP), however, this decline occurred at a much slower rate than in Malawi [6,7].

The genetic cause of CQR was found to be a single point mutation changing the amino acid lysine (K) to threonine (T) at position 76 in the gene coding for the *P. falciparum *CQ resistance transporter *(pfcrt) *[8-10]. This mutation arose only in a small number of founder locations, from where the resistant parasites spread worldwide. As a consequence, the variability of the chromosomal regions flanking the *pfcrt *gene on chromosome 7 is strongly reduced in resistant parasites, a phenomenon referred to as selective sweep [11]. The chromosomal haplotype - a characterization of this part of the chromosome, assessed either by SNP or microsatellite analysis - is, therefore, highly conserved in resistant parasites and specific for the respective founder locations. In contrast, sensitive parasites generally have very diverse haplotypes [11,12].

In Gabon, CQ use was officially discontinued in 2003 and replaced by the combination of artesunate and AQ. Before the change in national treatment guidelines the prevalence of the mutant *pfcrt *allele 76T was repeatedly measured to be 100% [13-15]. The goal of this study is to investigate if this change was associated with a return of the wild type *pfcrt *allele - as has been observed in other African countries - and if it affected genetic diversity in the surrounding chromosomal areas.

## Methods

### Study site and population

All samples were taken from clinical studies conducted at the Albert-Schweitzer-Hospital in Lambaréné, Gabon. Malaria in Lambaréné is hyperendemic with stable transmission throughout the year, the predominant species is *P. falciparum *[16]. Patients had been recruited in Lambaréné and surroundings (radius approx. 60 km). Informed consent had been obtained by all patients or their guardians and all studies had been approved by the Ethics Committee of the International Foundation of the Albert Schweitzer Hospital. For the 1995/96 cohort, samples from the 1/95C study were analysed [17]. The 2002 cohort consisted of samples from the 2002 case control study [15,18]. For the years 2005 - 07 samples were obtained from the Antigenic Diversity Study [19,20], the Ferroquine Tolerance Trial [21], the IPTi Study [22,23] and the SP Efficacy Trial [24].

### DNA extraction

Parasite DNA was extracted either from whole blood samples or from dried filter blood spots using the QIAamp^® ^DNA Mini Kit or DNA Blood Mini Kit (Qiagen, Hilden, Germany) according to the manufacturer's protocol. Extracted DNA was stored at -20°C.

### Polymerase chain reaction and sequencing

Polymerase chain reaction (PCR) was carried out using 2.5 mM MgCl_2_, 0.2 mM denucleoside triphosphates, 0.5 μM forward and reverse primer, 1.5 U Taq polymerase and 5 μl DNA in a total volume of 50 μl. In case of nested PCRs 5 μl PCR product of the first amplification step was used as template in the second step. PCR reagents were obtained from Qiagen, primers were ordered from Operon (Cologne, Germany). Reactions were run in a Biometra Uno II Thermal Cycler (Biometra, Goettingen, Germany), a MyCycler Thermal Cycler (Bio-Rad, Munich, Germany) and a MJ Research PTC-200 (GMI, Ramsey, USA). PCR products were stained with SYBR Green (Biozym, Hess. Oldendorf, Germany) and run on a 1.5% agarose gel. PCR products submitted to sequencing were cut out of the gel, extracted and purified using the NucleoSpin Extract II Kit (Machery-Nagel, Dueren, Germany) according to the manufacturer's protocol. This was followed by a sequencing PCR using 1/10 BigDye Terminator (Applied Biosystems, Warrington, UK) and approx. 1.5 ng DNA/100 bp. 25 cycles were run consisting of melting at 94°C for 10 sec, annealing at 50°C for 5 sec and elongation at 60°C for 4 min. Obtained PCR products were purified using Sephadex 96-well plates (GE Healthcare, Little Chalfont, UK) and sequenced by a Abi Prism 3000 Gene Analyzer (Applied Biosystems). Sequences were displayed with the BioEdit software (Ibis Biosciences, Carlsbad, USA).

### Detection of *pfcrt *mutations by restriction digest and sequencing

CQR of isolates was determined by detection of the mutant or wild type allele of the *pfcrt *gene at position 76. The CQR conferring mutation K76T as well as seven other resistance associated mutations (M74I, N75E, A220S, Q271E, N326S, I356T, R371I) were detected using a method consisting of a nested PCR followed by mutation specific restriction digestion as described by Djimde *et al *[25]. The mutations M74I and N75E were directly detected via PCR product sequencing.

### Haplotype analysis

The chromosomal haplotype was defined as the specific combination of the alleles of the following six microsatellite markers: PE14D (distance from the *pfcrt *gene -45 kb), B5M77 (-18 kb), 3E7 (-11 kb), 9B12 (+2 kb), 7A11 (+21 kb) and PE14F (+57 kb). Additionally, the microsatellite B5M47 (-0 kb) was analysed in several isolates of the years 2005 - 07 but not taken into account for the haplotype analysis. The position of the individual microsatellites relative to *pfcrt *is calculated based on their coordinates on Chromosome 7 of the 3D7 genome strain (http://www.plasmodb.org). These differ somewhat from the originally reported distances by Wootton *et al *[11]. Alleles were determined as differences in repeat length. The repeat sequence motifs of the different microsatellites were as follows: PE14D (TAC)/(TAA), B5M77 (TA), 3E7 (TA)/(T)/(TA), 9B12 (TA), 7A11 (TTATA), PE14F (TAA). In case of multiple infections only samples with a clear dominance of one microsatellite allele in the sequence ferrogram were included for further analysis [12].

Microsatellite markers were amplified via PCR and sequenced. Primers and conditions were adapted from Su *et al *[26] but resulted in suboptimal amplification in field isolates. Therefore conditions had to be modified for all microsatellites and in most cases a nested PCR approach had to be implemented (see Additional file 1). The strains 3D7, Dd2 and FCR3 were used as positive PCR controls. The consistency of primers and conditions was verified by sequencing of the PCR amplification products of the control strains and comparing the obtained microsatellite sequences with the known sequences deposited in the NCBI database, which always showed a perfect match of the repeat lengths.

Chromosomal haplotype studies were done on 171 resistant samples from the years 1995 - 2007. Successful sequencing of the full set of microsatellites was achieved in 132 samples. In 145 samples at least the inner four microsatellites (B5M77, 3E7, 9B12, 7A11) covering a chromosomal fragment of approximately 40 kb could be sequenced. These samples consisted of 43 samples from the 1995/96 cohort, 47 samples of the 2002 cohort and 55 samples of the 2005 - 7 cohort. All sequences were submitted to Genbank. Individual samples can be identified through their study specific label, consisting of a study specific combination of letters and an isolate specific running number, as indicated below: 1/95C study (M or S, followed by a number, i.e. M1), 2002 case control study (N followed by a number, i.e. N1), Antigenic Diversity Study (three individual letters, i.e. MOA), the Ferroquine Tolerance Trial (TDU or FED or TDR, followed by a number, i.e. TDU1), IPTi Study (IP followed by a number, i.e. IP1), SP Efficacy Trial (SP followed by a number, i.e. SP1). Genbank accession numbers are: 3E7 sequences: HQ417163 -HQ417327, 7A11 sequences: HQ417328 - HQ417497, 9B12 sequences: HQ417498-HQ417655, B5M77 sequences: HQ417656 - HQ417824, PE14D sequences: HQ417825 - HQ417986, PE14F sequences: HQ417987-HQ418152.

### *msp2 *genotyping

50 isolates from all four studies of the years 2005 - 07 were further characterized by sequencing of the central region of the *merozoite surface protein 2 (msp2) *gene. Primers and conditions for an allele specific nested PCR were as described by [27]. MSP2 1^st ^step: forward primer F1 ATGAAGGTAATTAAAACATTGTCTATTATA and reverse primer F2 ATATGGCAAAAGATAAAACAAGTGTTGCTG, 94°C for 5 min, followed by 30 cycles of 94°C for 10 sec, 57°C for 30 sec, 72°C for 30 sec, and a final extension step of 72°C for 2 min. MSP2 2^nd ^step FC allele: forward primer FC27 F2 GCAAATGAAGGTTCTAATACTAATAG and reverse primer FC27 R2 GCTTTGGGTCCTTCTTCAGTTGATTC, MSP2 2^nd ^step 3D7 allele: forward primer 3D7 F2 GCAGAAAGTAAGCCTTCTACTGGTGCT and reverse primer 3D7 R2 GATTTGTTTCGGCATTATTATGA. PCR was carried out using the following conditions 94°C for 5 min, followed by 40 cycles of 94°C for 10 sec, 57°C for 30 sec, 72°C for 30 sec, and a final extension step of 72°C for 2 min.

### Statistical analysis

Conservation of any marker or haplotype was defined as the prevalence of the dominant allele. Development of the degree of conservation over the years was analysed by logistic regression. A chi-square test was applied for assessment of significance. Allele diversity was described by the expected heterozygosity *H*_*e *_using the formula: *H*_*e *_*= *[*n*/(*n*-1)][1-Σ*p*_*i*_^2^] with *n *being the number of isolates and *p*_*i *_the frequency of the *i*th allele [28]. Development of *H*_*e *_over the years was analysed by linear regression, an F test was applied for assessment of significance. In all applied tests *p*-values < 0.05 were regarded as significant.

## Results

### Prevalence of the mutant and wild type *pfcrt *allele before and after the withdrawal of chloroquine

In order to display the prevalence of the mutant *pfcrt *allele after 2003, 144 samples from the years 2005 - 07 were screened for the CQR conferring mutant 76T *pfcrt *allele. The restriction digest revealed only one sample with a wild type 76K *pfcrt *allele, thus a genetically sensitive isolate, in 90 samples from the years 2005/06 (1%) and three isolates with the wild type 76K *pfcrt *allele in 54 samples from 2007 (6%) (of the wild type isolates three were monoinfections and one was a mixed infection). Overall the prevalence of the mutant 76T *pfcrt *allele remained at 97% after the withdrawal of chloroquine from national treatment guidelines. Previous analysis of 151 samples from the years 1995/96 and 2002 had shown a prevalence of 100% for the mutant 76T allele [13-15]. For this investigation an additional 64 samples of these studies were analysed. The mutant 76T *pfcrt *allele was detected in all samples. Thus, in total all 215 analysed samples from the years 1995/96 and 2002 carried the mutant 76T *pfcrt *allele, confirming the very high prevalence of the mutant *pfcrt *allele in this area prior to the change in national treatment guidelines (see Table 1).

**Table 1:** Prevalence of the mutant and wild type *pfcrt *allele before and after the withdrawal of chloroquine from national treatment guidelines (2003)

	1995-2002	2005-07
**no. of samples**	215	144

**no. with mutant**	215 (100%)	141 (97%)

**no. with wild type**	0 (0%)	4 (3%)

Mutant: *pfcrt *76 T

Wild type: *pfcrt *76 K

*Pfcrt *haplotype analysis was conducted in the three wild type isolates that were monoinfections and 48 randomly picked mutant isolates from the years 2005 - 7. Sequencing of the three monoinfections showed that all carried the original wild type *pfcrt *haplotype CVMNK at the codons 72-76 and AQNIR at the codons 220, 271, 326, 356, 371. The reappeared wild type *pfcrt *allele thus did not result from a simple back mutation from T to K at codon 76. Sequencing of 48 randomly picked mutant isolates from the years 2005-7 showed that they uniformly carried the CVIET haplotype at the codons 72-76.

### Chromosome 7 haplotype characterization of mutant and wild type isolates

**Figure 1:**
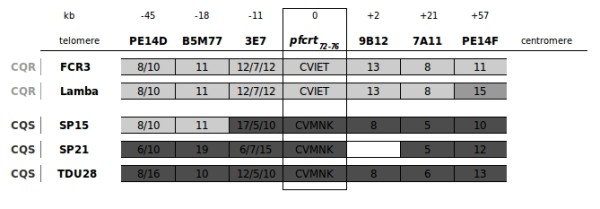
**Haplotype analysis of resistant and sensitive parasites**. The two most frequent local resistant haplotypes from the year 1995/6 are displayed. One of the haplotypes is identical to the FCR3 haplotype and represented 21% of all haplotypes analysed in 1995/6. The other haplotype (denoted "Lamba") represented 37% of all haplotypes in 1995/6. The haplotypes of the three sensitive monoinfections SP15, SP21 and TDU28 from the years 2005 - 7 are represented in the lower part of the figure. Alleles identical to the local resistant haplotypes are shaded in light grey, differing alleles in dark grey. Samples SP21 and TDU28 carry completely different chromosome 7 haplotypes, suggesting that they represent reintroduced sensitive parasites. In contrast SP15 carries the same alleles as the local resistant haplotypes at the microsatellite PE14D and B5M77 and subsequently starts to diverge. This suggests a backcross of the sensitive *pfcrt *allele. Sequencing of the marker 9B12 in the sensitive isolate SP21 was unsuccessful. Haplotypes are represented by indicating the number of repeats at each microsatellite. The *pfcrt *haplotype at codons 72-76 is also displayed.

To assess the effect of chloroquine pressure on the chromosomal haplotype, microsatellite allele analysis was initally performed on isolates from 1995/6. For characterization of the chromosome fragment surrounding the *pfcrt *gene six microsatellite markers were sequenced (PE14D, B5M77, 3E7, 9B12, 7A11, PE14F) covering a chromosomal fragment of approximately 100 kb (see Figure 1). Analysis of the obtained haplotypes revealed the presence of two most prevalent haplotypes in 1995/96. One haplotype was identical to the haplotype of the resistant lab strain FCR3 [11] (assumed to represent the dominant resistant haplotype in Africa and differing from Dd2 at the microsatellite 7A11) at all six analysed microsatellites and was found in 21% of the samples. The other haplotype, termed "Lamba", showed a different allele at the most centromeric marker PE14F (see Figure 1) and was prevalent in 37%. Together we defined these two haplotypes as the dominant local resistant haplotypes.

To assess whether the newly appeared *pfcrt *sensitive isolates from the years 2005-7 represented immigrated strains or local formerly resistant strains that had reacquired the wild type *pfcrt *allele, haplotype analysis of the wild type isolates was performed. Haplotype analysis was possible in the three monoinfections (samples SP15, SP21, TDU28) of the four wild type isolates. The isolates SP21 and TDU28 carried haplotypes with no resemblance to the dominant local resistant haplotypes. In contrast, the haplotype of isolate SP15 was identical to the dominant local resistant haplotypes at the most telomeric microsatellites PE14D and B5M77 and only started to diverge at microsatellite 3E7, approximately 11 kb upstream of the *pfcrt *locus (see Figure 1). This suggested that SP15 might have resulted from a backcross of a wild type *pfcrt *allele into a local resistant isolate.

### Prevalence of the dominant local resistant resistant haplotypes before and after the withdrawal of chloroquine

The prevalence of the dominant local resistant haplotypes was assessed in 1995/96 and 2002, before the withdrawal of chloroquine from national treatment guidelines, as well as in 2005-7. The dominant local resistant haplotypes maintained a high prevalence at all times although a decrease from 58% prevalence in 1995/96 to 31% in 2002 and 33% in 2005 - 07 (*p *= 0.01, see Table 2) was noticeable. However, it is striking that the observed development towards lesser conservation took place entirely between 1995/96 and 2002 - thus before the withdrawal of CQ - while no significant change in conservation could be observed between 2002 and 2005 - 07. Besides the dominant local resistant resistant haplotypes, multiple other individual haplotypes were identified. Most of these haplotypes, however, were found only once in the examined population. The total number of unique haplotypes present in the parasite population increased from 18 (including the two dominant haplotypes) in 1995/96 to 28 in 2002 and stayed at 28 in 2005 - 07.

**Table 2:** Prevalence of the local resistant haplotypes (100 kb) over the years

	1995/96	2002	2005-07
**no. of samples**	38	45	49

**no. of samples with local haplotypes (~100 kb)**	22 (58%)	14 (31%)	16 (33%)

**no. of samples with other haplotypes (~100 kb)**	16 (42%)	31 (69%)	33 (67%)

To assess genetic change in the chromosomal areas immediately adjacent to the *pfcrt *locus a local "inner" haplotype was defined as the chromosomal fragment defined by the four microsatellites B5M77, 3E7, 9B12, 7A11, covering a region of approximately 40 kb around the *pfcrt *locus. This revealed that in 1995/96 79% of the analysed isolates carried one identical haplotype in this chromosomal region. In analogy to the six-microsatellite-haplotype, the prevalence of this dominant local "inner" haplotype remained relatively high throughout the years, but showed a statistically significant decrease to 62% in 2002 and 58% in 2005 - 07 (*p *= 0.03, see Table 3). Again, the decrease in conservation was most pronounced between 1995/6 and 2002, before the change in national treatment guidelines.

**Table 3:** Prevalence of "inner" haplotype (40 kb) over the years

	1995/96	2002	2005-07
**no. of samples**	43	47	55

**no. of samples with local "inner" haplotype (~40 kb)**	34 (79%)	29 (62%)	32 (58%)

**no. of samples with other "inner" haplotypes (~40 kb)**	9 (21%)	18 (38%)	23 (42%)

To evaluate the propensity for genetic change at the individual microsatellite markers, the change in conservation from 1995/6 to 2005 - 07 was evaluated. This revealed a significant decrease in conservation at the two most centromeric microsatellites 7A11 (*p *= 0.04) and PE14F (*p *= 0.02) while the other microsatellite markers remained conserved.

**Figure 2:**
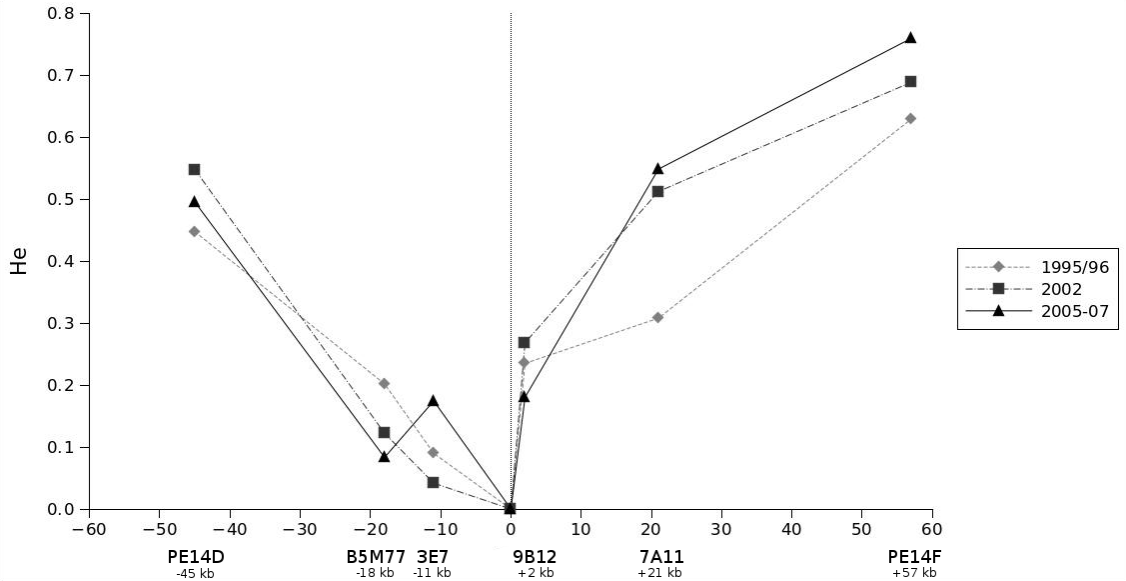
**Expected heterozygosity around the *pfcrt *locus before and after the removal of chloroquine from national treatment guidelines**. The expected heterozygosity *H*_*e *_is shown for each microsatellite in the resistant populations of the years 1995/96 (diamonds), 2002 (squares) and 2005-07 (triangles). On the x-axis the distance [kb] from the *pfcrt *locus is given. During all time periods *H*_*e *_increased with increasing distance from the *pfcrt *locus but was higher at the centromeric microsatellites compared to the telomeric microsatellites.

In order to assess the degree of genetic diversity at the individual microsatellites over the years, their expected heterozygosity *H*_*e *_during the different time periods (see Figure 2) was determined. As expected, *H*_*e *_was smallest at the microsatellites directly adjacent to the *pfcrt *locus and largest at the most distant microsatellites. During all time periods *H*_*e *_increased with increasing distance from the *pfcrt *locus. Furthermore, *H*_*e *_was higher at the centromeric microsatellites 9B12, 7A11 and PE14F compared to the telomeric microsatellites 3E7, B5M77 and PE14D, again suggesting a higher degree of genetic variability in the centromeric microsatellites. A trend towards increasing diversity over time was apparent for the two most centromeric microsatellites 7A11 and PE14F, however this was not statistically significant.

### Genetic diversity at the *msp2 *gene

The analysis suggested that the genetic sweep on chromosome 7 persisted in the local parasite population despite the removal of CQ from the national treatment guidelines. To estimate the potential diversity in the Lambaréné parasite population, the expected heterozygosity at the locus coding for MSP2, a surface antigen with a presumably high diversity was also determined. The *msp*2 locus from 50 isolates of the years 2005 - 07 was PCR amplified and the respective 3D7 (30) and FC27 (40) fragments were sequenced. A total of 48 different alleles were identified. The *H*_*e *_for the *msp2 *locus was calculated to be 0.98. In comparison, the mean genomic *H*_*e *_of African *P. falciparum *parasites was assessed to be 0.76 - 0.8 [28]. Of the microsatellites analysed in the 2005 - 07 population only the least conserved marker PE14F had a *H*_*e *_within that range (0.76). Contrasting, the microsatellite B5M47 lying directly adjacent to the *pfcrt *locus had a *H*_*e *_of 0.11 (see Table 4). Taken together, this shows that the potential for genetic diversity is very high in this population and highlights the high degree of genetic conservation surrounding the mutant *pfcrt *gene on chromosome 7.

**Table 4:** Expected heterozygocity of B5M47 and PE14 F on chromosome 7 and expected heterotzygocity of the *msp 2 *locus on chromosome 2

	B5M47	PE14F	*msp2*
**no. of samples**	54	69	50

**no. of alleles**	3	9	48

**H**_**e**_	0.11	0.76	0.98

## Discussion

This is the first report analysing the effect of the withdrawal of CQ from the national treatment guidelines in 2003 on the prevalence of the mutant *pfcrt *allele in Lambaréné, Gabon. Prior to the change in national treatment policy the prevalence of the mutant *pfcrt *allele 76T had been 100% [13,14]. However, even in the years 2005 - 07 the level of CQR was still as high as 97%. Thus the decrease of CQR after discontinuance of CQ use observed in Gabon is much smaller compared to the development in Malawi where resistance decreased within eight years from 85% to 13% [3,4], as well as compared to other African countries such as Kenya [7] and Tanzania [6] where recent reports also documented a decrease in CQR from 95% to 60% and 94% to 70% respectively, after national CQ stops. What explanations can be invoked to explain the differences in the decline of CQR in these countries and Gabon?

The resurgence of CQS in Malawi, Kenya and Tanzania was observed after changes in national treatment guidelines from CQ to SP. In contrast, Gabon changed its national treatment guidelines from CQ to the combination of artesunate plus AQ. AQ is structurally related to CQ with both belonging to the group of 4-aminoquinolines. A recent report from Ghana analysed the prevalence of the mutant *pfcrt *allele after a change in national treatment guidelines from CQ to artesunate and AQ. The authors observed a decrease in the prevalence of the mutant *pfcrt *allele only in the northern part of the country, raising the question if this effect truly represented a national trend [29]. In contrast, a recent report from the Gabonese region of Franceville showed no change in the prevalence of the mutant *pfcrt *allele after the change in national treatment guidelines [30]. Data from clinical trials and the 7G8xGB4 and HB3xDd2 genetic crosses clearly suggest that AQ strongly selects for the resistance conferring mutant *pfcrt *allele [31,32]. Analysis of progeny of the 7G8xGB4 genetic cross showed that the South American *pfcrt *haplotype SVMNT at the codons 72-76 is associated with high level resistance against monodesethylamodiaquine, the active metabolite of AQ. Reports from Angola [33] and Tanzania [34] have shown the presence of the SVMNT *pfcrt *haplotype following the use of artesunate and AQ or the single use AQ, suggesting that AQ may select for this haplotype [35]. However, none of the 48 randomly sequenced mutant *pfcrt *alelles from the years 2005 - 07 carried the SVMNT haplotpype in this analysis of the Lambaréné parasite population. In view of the high conservation of the chromosomal regions flanking the *pfcrt *gene even in 2005 - 07 this may simply reflect the continued selection of the most abundant *pfcrt *haplotype CVIET. The introduction of the SVMNT haplotype may thus be influenced by the specific characteristics of local parasite populations. Taken together the data suggest that in Gabon the introduction of artesunate plus AQ may have selected for continued conservation of the mutant *pfcrt *locus.

Interestingly, a decrease in conservation of the local resistant chromosomal haplotypes was observed between 1995/96 and 2002, prior to the removal of CQ from the national treatment guidelines. A similar phenomenon was observed in Kenya, where CQR started to decline prior to the official change in national treatment guidelines, possibly as a consequence of declining use due to decreased clinical effectiveness. Similarly, local treatment guidelines at the Albert Schweitzer Hospital were already changed in 1994 - from CQ to SP. A decrease in CQ pressure might thus have contributed to more genetic diversity surrounding the *pfcrt *locus, however the resurgence of CQS might have been precluded due to the continued use of CQ in the surrounding areas. This is supported by an overall increase of different resistant haplotypes from 1995 to 2002 and by the decrease in prevalence of the most common "local resistant haplotypes".

What could be the mechanism that led to the reappearance of the wild type *pfcrt *allele? None of seven point mutations in the *pfcrt *gene commonly found in CQR strains from Africa and Asia [11,36] were present in any of the genetically sensitive isolates, who thus appear to be genuine wild type *pfcrt *alleles. This rules out the possibility that the sensitive isolates originated from a simple back mutation at the crucial position 76 of the *pfcrt *gene in former resistant parasites and is consistent with the results of others [3-5]. The implication of this finding is that the wild type *pfcrt *allele must have been reintroduced into the current population through import from another geographic area, or by reexpansion of sensitive parasites that were under the detection level of previous investigations. However, given the persistently high prevalence of the resistant haplotypes the former possibility appears more likely. Chromosome 7 haplotype analysis of the three sensitive monoinfections showed no conservation of microsatellite alleles. Comparison to the local resistant population showed that one (isolate SP15) shared the two microsatellites PE14D and B5M77 at the most telomeric end of the analysed haplotype with the dominant local resistant haplotypes. To estimate the likelihood that the SP15 haplotype resulted from a cross of a local resistant and a sensitive isolate, the number of resistant parasites with the same PE14D and B5M77 alleles as SP15 was quantified. Of the 172 haplotypes analysed in the study, 107 carried the exactly same PE14D-7A11 microsatellite alleles as SP15, suggesting that SP15 might represent a genetic cross between a sensitive strain and a formerly resistant strain of the local population.

## Conclusions

The data presented in this manuscript show that four years after the discontinuance of CQ as first-line therapy in Gabon the prevalence of the mutant *pfcrt *allele is still extremely high in the Lambaréné area. This finding stands in marked contrast to a more pronounced development towards CQS in some other African countries. One possible explanation may be an ongoing selection for the mutant *pfcrt *allele by AQ as part of an artemisinin-based combination therapy. It has repeatedly been proposed that CQ may return to clinical use in the future. The results of this investigation and the result of others [6,7] sound a note of caution and clearly show that this can only be contemplated in areas where the return of CQ sensitivity has clearly been demonstrated by molecular drug sensitivity monitoring.

## Competing interests

The authors declare that they have no competing interests.

## Authors' contributions

MF conceived the study and designed the experiments, NL carried out the molecular genetic studies on microsatellites and on the *msp2 *gene, PM carried out part of the *msp2 *analysis and extracted the DNA of part of the 1995/6 and 2002 samples, FN supervised part of the *msp2 *analysis, DUK and MDB conducted the Antigenic Diversity Study, JG, GMN, MG conducted the IPTi study and the SP Efficacy trial, GMN, CS, BL conducted the Ferroquine tolerance trial, KD performed the statistical analysis, PK coordinated the study and provided conceptual advice. All authors read and approved the final manuscript.

## Supplementary Material

Additional file 1 **Primers and conditions for microsatellite PCR amplification**. Table of primer sequences and PCR cycling conditions employed for microsatellite analysis Click here for file
