# Supplementary material for: A thirteen-year analysis of Plasmodium falciparum populations reveals high conservation of the mutant pfcrt haplotype despite the withdrawal of chloroquine from national treatment guidelines in Gabon
Source: Malar J. 2011 Oct 17;10:304. doi: 10.1186/1475-2875-10-304 (PMC3215978; doi:10.1186/1475-2875-10-304)
Supplement: Additional file 1 — Primers and conditions for microsatellite PCR amplification. Table of primer sequences and PCR cycling conditions employed for microsatellite analysis [file 1475-2875-10-304-S1.DOC]

Additional file 1. Primers and conditions for microsatellite PCR amplifications

| Microsatellite | step | Forward primer sequence | Reverse primer sequence | Initial  denaturation | denaturation | annealing | extension | Final  extension | cycles |
| --- | --- | --- | --- | --- | --- | --- | --- | --- | --- |
| PE14D | 1 | GGATATTATGATTCAATAAATC | GATCATTTGCATGACTAGC | 94°C / 2 min | 94°C /  20 sec | 58°C /  20 sec | 60°C /  30 sec | 60°C /  2 min | 30 |
|  | 2 | TGTAATGAATGATTCTAATACCAC | TTGGACCATGCTTCACAG | 94°C / 2 min | 94°C /  20 sec | 58°C /  20 sec | 60°C /  30 sec | 60°C /  2 min | 30 |
| B5M77 | 1 | TAAAGTCTTTCAATACATATG | GAAATAATTTCATATACACAC | 94°C / 2 min | 94°C /  20 sec | 51°C /  20 sec | 60°C /  30 sec | 60°C /  2 min | 45 |
| 3E7 | 1 | GATCGAAGTACAGATAGAAT | GTGCAAGATATAATAAAAGTTG | 94°C / 3 min | 94°C /  30 sec | 56°C /  30 sec | 60°C /  3 min | 60°C /  10 min | 30 |
|  | 2 | AAGAATGAAAGTATTTTTAGC | CCCCTTCAAAAAGGAAATAACAC | 94°C / 2 min | 94°C /  20 sec | 56°C /  20 sec | 60°C /  30 sec | 60°C /  2 min | 30 |
| B5M47 | 1 | ATGACGAGCGTTATAGAGAA | CAAAACTATAGTTACCAATTTTG | 94°C / 3 min | 94°C /  30 sec | 48°C /  30 sec | 60°C /  1 min | 60°C /  3 min | 45 |
| 9B12 | 1 | ATATATTCCAGTATGTTCGC | AATGATACAATGGGATTTAC | 94°C / 2 min | 94°C /  20 sec | 51°C /  20 sec | 60°C /  30 sec | 60°C /  2 min | 45 |
| 7A11 | 1 | GTAACTGTAATGTGTACTTG | CTCTTCTCTGAGTAAATATG | 94°C / 3 min | 94°C /  30 sec | 53°C /  30 sec | 60°C /  1 min | 60°C /  3 min | 30 |
|  | 2 | ATGTGTAAGGAGATAGTATA | CAACTTTCTCTTTTTAAATATTAC | 94°C / 2 min | 94°C /  20 sec | 56°C /  20 sec | 60°C /  30 sec | 60°C /  2 min | 30 |
| PE14F | 1 | GCTGGAACTAATTGTTTGT | AGTATGAACATTTTGTACAT | 94°C / 2 min | 94°C /  20 sec | 58°C /  20 sec | 60°C /  30 sec | 60°C /  2 min | 30 |
|  | 2 | CTGTGGATAATGATATTC | GTCCATTGAAAAGATAGG | 94°C / 2 min | 94°C /  20 sec | 58°C /  20 sec | 60°C /  30 sec | 60°C /  2 min | 30 |
